# The Treatment of Wounded Joints

**Published:** 1901-10-26

**Authors:** 


### THE TREATMENT OF WOUNDED JOINTS.

At the last meeting of the Clinical Society the treatment of wounded joints was discussed by Mr. Cuthbert Wallace, who related a series of cases. He held that the cleansing of such wounds must be mechanical, that all dirty tissues should be cut away with knife or scissors, and that a douche, if used, was only beneficial because it removed fragments of dirt or soiled tissue; it was therefore sufficient to employ sterilised saline fluid, and antiseptic solutions were unnecessary. A search for a wound into a joint was held not to be desirable as, if it should be found, it was unlikely that much good could be done, while if an opening should be accidentally made into the capsule serious injury might result. On the other hand if the opening into the joint were obvious it would be well to close the capsule, without irrigation of the joint, and to await developments. In cases of punctured wounds it was thought that the best treatment was arthrotomy and suture of the joint cavity, using sterilised saline solution. This process could be repeated once or twice if the condition required it, but if after this the temperature still remained high the incision into the joint could be left open and lavage practised daily. Drainage by means of tubes through the joint, or continuous irrigation were, he thought, likely to do more harm than good. In discussing this paper the president (Mr. Arthur Barker) agreed that in these cases antiseptics were not only useless but often positively injurious. For many years he had employed only irrigations of normal saline, and under this method his results had improved.

